# A scoping review on developmental activities of girls' and women's sports

**DOI:** 10.3389/fspor.2022.903886

**Published:** 2022-09-23

**Authors:** Carrie M. Peters, David T. Hendry, Nicola J. Hodges

**Affiliations:** ^1^Motor Skills Lab, School of Kinesiology, University of British Columbia, Vancouver, BC, Canada; ^2^Department of Sport, Exercise and Rehabilitation, Northumbria University, Newcastle upon Tyne, United Kingdom

**Keywords:** expertise, deliberate practice, talent, play, specialization

## Abstract

We provide a scoping review of research on athlete development in girls' and women's sports. Our emphasis is on pathways to expertise in the context of deliberate practice theory and associated models, such as the Developmental Model of Sport Participation (DMSP). Despite rationale for sex and gender differences in sport development, there are relatively few studies where the developmental pathways of female elite athletes have been evaluated. We sought to map the scope of the literature on this population over the last 30 years, focusing on measures of practice types and amounts. Following an extensive search of the literature, 32 studies were identified that included all female participants or presented sex/gender disaggregated data. Retrospective methods were commonly used to quantify practice, play and specialization. National-level athletes were the most represented, although there was considerable heterogeneity in sport and expertise-level, making general or comparative judgements challenging. We identified some groups that had accumulated high volumes of practice at a young age, particularly in soccer and gymnastics. Across sports and studies, early majority hours of engagement in the primary sport was the norm. Athletes deviated from predictions in the specialization pathway detailed in the DMSP, by continuing to participate in other sports throughout childhood and adolescence. In addition to highlighting the relative paucity of data pertaining to athlete development pathways in female athletes, we show that the data from these groups deviate from predictions detailed in current models of athlete development.

## Introduction

There is a substantial body of literature on the topic of “talent” development in sport and specifically on the question of how sport-specific practice amounts and types, as well as age of engagement, impact on attainment of sport expertise [e.g., [1–3]]. In most of the work on this topic, the developmental experiences of male participants have been the majority focus, or the data has been aggregated across males and females. Because models of expertise development are dependent on these studies, these models are likely to be skewed in their descriptions and hence predictions about pathways to expertise in women's sports. This knowledge gap was highlighted in a recent scoping review of talent development in sports [1], where only ~10% of the included studies focused solely on female athletes (~45% were exclusively on male participants, ~30% included both male and female participants and ~15% of studies did not report the sex/gender of the participants). In this scoping review, we aim to assess and evaluate the current knowledge of the pathways that are associated with expertise in youth and adult high-level female athletes.

Compared to boys, girls mature biologically and reach ages of peak motor skill development earlier [4, 5]. Girls are also less likely to engage in sport and physical activity [6–8] and have fewer opportunities for elite performance [such as paid professional sport leagues [9, 10]]. Some athlete development frameworks include gender specific age ranges for training based on gender differences in peak height velocity [11], where girls reach peak height velocity earlier than boys. Because of these biological differences, interpretations and definitions of early specialization should differ for female and male athletes. Lima et al. suggested that the early specialization of female athletes may be somewhat protective, allowing for better adjustment of athletic performance during pubertal changes [12]. Female athletes also show differences in their response to training and to other psychosocial factors, such as their relationships with parents and peers [13], leading us to expect that the developmental pathways to attain expertise may differ between male and female athletes.

In support of the idea that sex/gender impact developmental pathways, differences have been identified in reviews of the relative age effect [14–17]. Girls and women do not always exhibit the same advantage for birth month as their male counterparts. Differences have been attributed to biological maturation and socialization factors, where post-pubescent female characteristics (such as shorter legs and wider hips) constrain the athletic development of athletes that mature early (or those born earlier in the year) and social values and norms can deter early maturing female athletes from pursuing sport competitively [16]. Moreover, in a review of specialization and diversity in sport, gender was noted as a potential moderator of early specialization with some evidence that girls specialized more than boys [2]. However, in only a third of the studies included in this earlier review [2] was specialization defined, primarily based on a descriptor of intense engagement in year-round practice within one sport at the exclusion of others.

Here we discuss sex/gender as a binary concept, focusing on demographically described female participants in research publications. We acknowledge that gender exists on a spectrum and that the experiences of non-binary athletes have been omitted in the current body of literature. We would like to highlight that the terms sex and gender are also not interchangeable, with the former referring to biological and genetic differences and the latter to the roles and relationships ascribed by society [18]. Both sex and gender factors likely influence the developmental pathways of female athletes, but we are unable to disentangle the two here.

A key facet of the development of sport expertise is the accumulation of deliberate practice activities [19, 20]. According to deliberate practice theory, there is a monotonic relationship between the time engaged in deliberate practice and the level of performance. Deliberate practice is characterized as being effortful, relevant to performance goals, individualized, coach-led, not inherently enjoyable, and has a feedback component [for a recent sport-focused review see [21]]. Originally grounded in music development, there has been some debate as to whether the tenants of deliberate practice, specifically the monotonic benefits assumption, can be generalized to sporting contexts [22–24]. However, studies comparing practice histories of skilled and less skilled athletes have shown that the former accumulate more hours in deliberate practice activities across a range of sports and in a somewhat monotonic fashion [25–27].

Approximately a decade after deliberate practice theory was proposed, Côté et al. published work on the Developmental Model of Sport Participation (DMSP), which had two pathways leading to sports expertise [28–30]. Based on deliberate practice theory, an early specialization pathway was outlined to include high amounts of practice accumulated from a young age in one sport. In contrast, the early sampling pathway was characterized by early multi-sport involvement, high amounts of play and with later specialization in adolescence. In this second pathway, the sampling years (6–12 yr) were characterized by high volumes of play and participation in many sports. Specialization would not begin until the adolescent years (13–15 yr), where athletes focus on one or two sports and engage in equal amounts of practice and play activities. In the investment years (16–18 yr), athletes increase commitment to one sport and engage in a high volume of deliberate practice [30, 31]. The accumulation of diversified sport experiences and play in the early sampling pathway was thought to encourage the broad development of physical and psychosocial skills that benefit future athletic development [32]. In contrast, early specialization was thought to benefit the attainment of sports expertise only when peak performance occurs at a young age, such as in gymnastics and figure skating [33, 34].

Although there has been support for some of the predictions emanating from the DMSP, there have been issues in defining specialization and hence determining pathways based only on two categorically distinct pathways [35, 36]. Single sport participation, high amounts of deliberate practice, year-round training, exclusion of other sports, and intense training have differentially been used as criteria of specialization across studies [37, 38].

In samples of male soccer players, athletes participated in high volumes of sport specific practice and play at relatively young ages, consistent with the early specialization pathway, but diverged from predictions in that pathway by also sampling other sports in childhood [39–42]. Thus, a third pathway has been proposed, characterized by early majority engagement in the primary sport, without limiting participation in other activities [40]. This third pathway may offer some “protection” against proposed negative motivational consequences from high amounts of practice, such as burn-out and low intrinsic motivation [43–45].

A provocative idea is that differences in childhood activities which lead to later success take time to show up and hence distinguish across athletes that attain juvenile vs. adult success [46, 47]. As such, different pathways might characterize the development of adult and adolescent expertise [46]. Indeed, a meta-analysis across multiple sports showed that athletes who attained international success as junior athletes showed a more specialized pathway compared to national or regional athletes, whereas this pattern was reversed for world class adult elite athletes [47]. There was more multi-sport participation during development among adult world class elite athletes compared to lesser skilled peers. Although these data are correlational, based on cross-sectional comparisons and aggregated across various sports with different participation rates and ages of peak performance, success at junior levels is not a good predictor of adult success [48] and ~25% of athletes who attained elite performance as adults in a study of Portuguese athletes did not compete internationally in their youth [49].

In this scoping review, we detail the research (1990 to May 2021) specifying activities undertaken by female “elite” athletes during childhood. In addition to collecting practice, play, and specialization measures, we present key study characteristics and participant demographics to contextualize the results and to highlight gaps in study populations. We sought and evaluated studies that captured female athletes competing at relatively high levels of performance, in both junior and adult groups. Our main aims were to synthesize the data pertaining to childhood activities and demographics of adult and youth female elite athletes to describe and evaluate pathways to expertise in view of current research and models of athlete development.

## Methods

### Study search and screen

The protocol was set a priori in accordance with current best practices for scoping reviews [50, 51]. To inform the search strategy, we reviewed known studies examining girls' and women's sports' participation and scoping and systematic reviews in the field. The primary search was conducted by the first author in SPORTDiscus to identify studies published between January 1990 and May 2021. We chose this period as it slightly pre-dates the seminal study on deliberate practice by Ericsson et al. [20], which resulted in a high volume of research in sport related to pathways of skill development and measures of practice. Boolean search terms were used to combine subject terms and synonyms broadly encompassing the population (high level female/women athletes) and the outcomes of interest (practice, participation, specialization/diversification, and developmental activities). The search was limited to scholarly articles and studies where an English abstract was available. Further searching was done in Google Scholar and manual searching of the reference lists of included studies and several key review papers and books. We also conducted a forward search by reviewing studies that cited the included studies and prominent review papers. The primary search uncovered studies that included both male and female athletes. If there was gender disaggregated data, these were included.

Study screening was done through Covidence (Covidence systematic review software, Veritas Health Innovation, Melbourne, Australia. Available at www.covidence.org). The first author conducted an initial title and abstract screen of the identified studies and the full text screen of the studies selected in the title and abstract screen. An independent reviewer (author two) screened 20% of these studies at both stages. Discrepancies in the title and abstract screen were automatically sent to the full text review and discrepancies in the full text screen were resolved through discussion with all three authors.

Empirical studies were included for review if they included measures of practice, play, multisport participation, or specialization. The scope was limited to capture a relatively elite sample of adult and youth female athletes. We acknowledge that there are issues in defining elite status and to maintain transparency in our definitions we keep descriptors in our analysis to allow better inferences as to “elite” status [e.g., [52–54]]. With respect to inclusion based on “elite” status (as detailed in the data extraction section below), we sought studies with adult participants who were competing at the Varsity/University level or higher, including National team athletes and premier/professional league athletes. For youth (U18) elite athletes, we restricted inclusion to athletes that were part of a national training squad or competing internationally. Studies that did not include participant gender in the abstract were sent to full text review, leading to a large number of studies (*n* = 252) screened at the full-text level. When gender was not reported, these studies were excluded. We included all study design-types, including retrospective, cross-sectional and longitudinal. In some studies, variables of interest, such as practice hours or years of engagement, were reported as demographic measures in the participant's section and not outcome measure. These studies were not included as measures were often poorly defined, leading to ambiguity in how they were obtained.

### Data extraction

Results were extracted by the first author using a custom spreadsheet piloted by the first two authors. The second author audited the data extracted from a random sample of 20% of the included studies and no discrepancies were identified. We recorded study details such as the study design, the country in which the data were collected and the sport. We recorded how the authors described the athletes' expertise and categorized them into groups. Adult athletes were categorized by us as “Super Elite” if they were medalists at international level competitions or were ranked highly internationally (i.e., within the top 10). Adult athletes were labeled “National” if they represented their country at an international level of play but did not meet the criteria for Super Elite. Adult athletes that played for a university or college athletic program were classified as “Varsity.” We did not distinguish between programs competing at different levels of university competition. Athletes that played their sport as a career and were paid as full-time players were classified as “Professional” and those that played for a high-level club in their country (e.g., premier league) but did not meet any of the above criteria were classified as “Elite Club” (this could include semi-professional athletes). Because in women's sports there are few opportunities for professional play [55], resulting in a paucity of groups in our sample at this level, we collapsed across the Elite Club and Professional categories and termed all as “Elite Adult” in the reporting of data. Groups that comprised athletes who were under 18 years but were competing at an international level or training with a national development squad were included in our analyses and classified as “Youth Elite.” Therefore, for descriptive comparisons of the groups, we consider the order of Super Elite, National, Elite Adult, Varsity, and Youth Elite as most expert (Super Elite) to least expert (Youth Elite).

Because we were interested in potential factors leading to adult expertise, we did not include youth or adult club-level athletes (including those competing at a provincial/state level). Participants' current age and the age when measures were taken were also recorded as were definitions of all dependent variables related to time spent in practice and play activities and sport specialization/diversification. When data were presented in figures in extracted studies, a plot digitizer tool (WebPlotDigitizer, https://automeris.io/WebPlotDigitizer) was used to extract numeric values.

### Data aggregation and transformations

We reported measures of practice, multisport participation, specialization and play as a function of sport in childhood (6–12 yr), early adolescence (13–15 yr) and late adolescence (16–18 yr) years, corresponding to the sampling, specializing and investment years, respectively [29]. In dividing age categories in this way, rather than presenting yearly amounts at each age, we were able to include more studies within an age category and better synthesize the data. Such dividing of data across these age categories also allowed us to compare the data to existing models of athlete development [30, 40]. When data were reported across multiple categories (e.g., from ages 14–18 yr), intermediate categories were added spanning the age periods (such as “sampling and specializing”). When authors reported multiple data points within a category of the DMSP (i.e., reported by age), data were averaged or summed depending on the measure (i.e., averaged for hours/year and summed for aggregated hours). When researchers reported data as a proportion of participants (e.g., 50% of the sample started their sport before the age of 10 years), we calculated and reported the median response. In cases where authors reported total years of involvement in the sport rather than start age of primary sport involvement, we subtracted the years of involvement from the current reported age of participants.

## Results

### Included study characteristics

**Figure 1:**
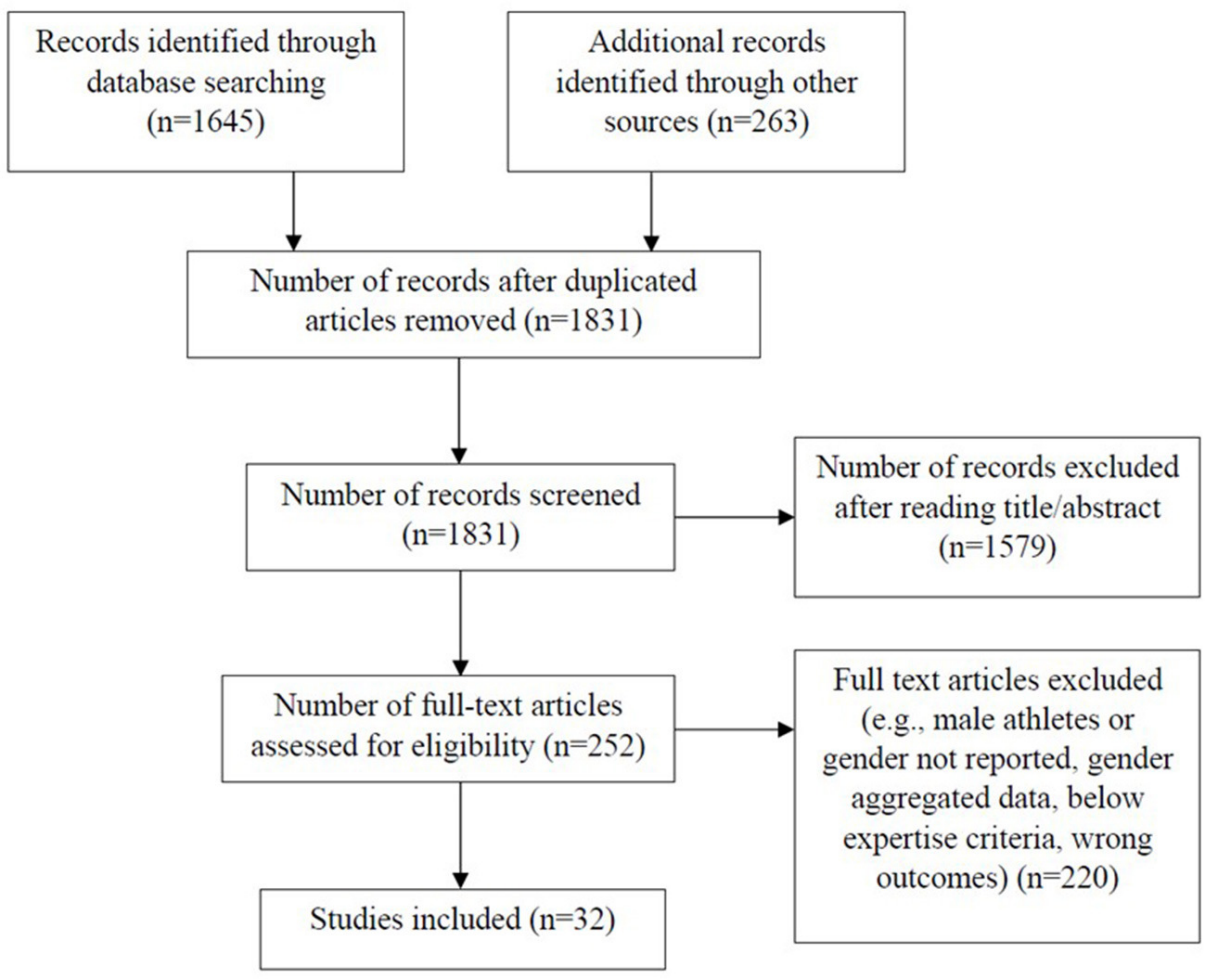
PRISMA flow diagram for the scoping review process.

From the 1,831 papers identified, 32 studies met the inclusion criteria and were included in our analyses as detailed in the PRISMA flow diagram in Figure 1. Of this final set of studies, group level data were extracted (*n* = 42 groups). In 11 cases, data from multiple groups representing different levels of expertise and in some cases different sports were extracted from the same study publication. In determining groups, we noted that some independent publications were based off the same group sample, such that there was the possibility of duplicate reporting of dependent variables. Therefore, although we include all studies in our reporting of study characteristics, we removed duplicate dependent variables in our reporting of outcomes^1^. Table 1 gives details of all included studies including sport, expertise classification, the country where data were collected, group sample size, and outcome measures reported (i.e., practice, play, multisport participation, and specialization).

**Table 1:** Included study characteristics.

**Study**	**Country**	**Sport**	**Expertise**	**N**	**Outcome domains**
Baker et al. [63]	Australia	Variety^a^	National	7	Practice, other sport participation
Baker et al. [64]	Germany	Handball	Youth Elite	45	Practice, other sport participation
Barynina and Vaitsekhovskii [65]	USSR	Swimming	National	Not reported	Specialization
Bjørndal et al. [66]	Norway	Handball	Youth Elite	21	Practice, specialization, other sport participation
Blijlevens et al. [67]	The Netherlands	Gymnastics	National	6	Practice
			Youth Elite	4	
Bruce et al. [68]	Australia	Netball	National	19	Practice, play, specialization, other sport participation
			Youth Elite	20	
Buckley et al. [69]	USA	Variety^b^	Varsity	331	Specialization
Coutinho et al. [70]	Portugal	Volleyball	Professional	35	Practice, other sport participation
Coutinho et al. [56]	Portugal	Volleyball	National	15	Practice, specialization, Other sport participation
Coutinho et al. [57]	Portugal	Volleyball	National	15	Play, other sport participation
Coutinho et al. [71]	Portugal	Volleyball	National	15	Play, other sport participation
Cowan et al. [59]	USA	Alpine ski	Youth Elite	91	Practice, play, other sport participation
da Matta [72]	Brazil	Volleyball	Super Elite	10	Practice
			Varied^f^	10	
de Bosscher and de Rycke [73]	Multiple locations	Variety^c^	National	1,253	Practice
DeCouto et al. [60]	USA	Alpine ski	Youth Elite	45	Practice
Duffy et al. [74]	Not reported	Darts	Super Elite	6	Practice, play
Fawver et al. [58]	USA	Alpine ski	Youth Elite	88	Practice, play, other sport participation
Ford et al. [75]	Multiple locations	Soccer	National	86	Practice, play, specialization, other sport participation
Güllich [76]	Germany	Soccer	National	14	Practice, specialization, other sport participation
			Professional	15	
Hendry et al. [77]	Canada	Soccer	National	21	Practice, play, other sport participation
			Varsity	24	
Hodges et al. [78]	Canada	Triathlon	Varied^g^	17	Practice
		Swimming	Elite Club	28	
Hodges et al. [79]	Canada	Triathlon	Varied	17	Practice
Hume et al. [80]	New Zealand	Rhythmic Gymnastics	National	5	Practice, other sport participation
			Youth Elite	25	
Johnson et al. [61]	USA	Swimming	Super Elite	4	Practice, other sport participation
			Varsity	3	
			Youth Elite	2	
Johnson et al. [62]	USA	Swimming	Super Elite	3	Practice
			Varsity	2	
Law et al. [33]	Canada	Rhythmic Gymnastics	Super Elite	6	Practice, specialization, other sport participation
			National	6	
Leite and Sampaio [81]	Portugal	Basketball	National	132	Practice, other sport participation
Naisidou et al. [82]	Greece	Handball	Youth Elite	24	Practice
Post et al. [83]	USA	Variety^d^	Varsity	115	Specialization
Staff et al. [84]	UK	Track & Field	National	28	Practice
Storm et al. [85]	Denmark	Variety^e^	National	10	Specialization, other sport participation
Timmerman et al. [86]	Australia	Field Hockey	Youth Elite	18	Practice, play, other sport participation
			Youth Elite	24	
			Youth Elite	9	

^a^Netball and field hockey.

^b^Collected from a variety of sports.

^c^Collected across 37 different sports.

^d^Basketball, golf, ice hockey, soccer, tennis, softball, and volleyball.

^e^Handball, orienteering, soccer, kayaking, rowing, sailing, swimming and golf.

^f^Five athletes were from club and recreational levels, and five were playing at the Varsity level.

^g^Recruited from a highly ranked club; 5 athletes had competed at the world championship level, 4 at the national level, 1 at the provincial level and 7 at the local level.

### Practice

**Figure 2:**
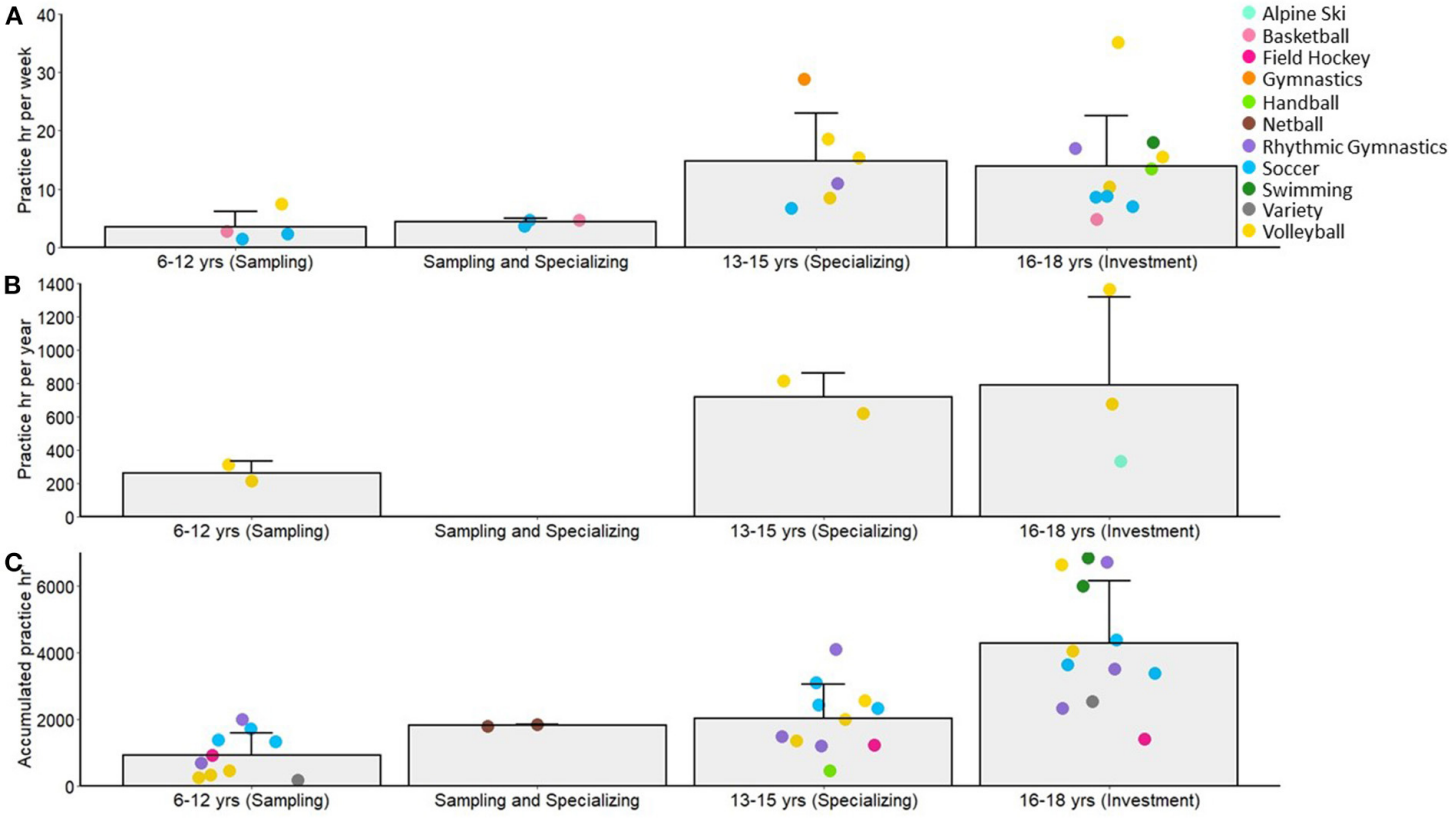
Sample (bar) and individual study means (colored circles) across different age categories defined in past literature as the sampling, specializing and investment years, for practice hours per week **(A)**, practice hours per year **(B)**, and accumulated practice hours **(C)**. Error bars represent sample standard deviation, the intermediate category (Sampling and Specializing) contains data that were reported across ages spanning the sampling and specializing years.

Twenty-two studies included broad measures of practice. In Figures 2A–C, respectively, we present practice hours per week, practice hours per year, and accumulated practice hours across what have been termed the sampling years (6–12 yr), the specializing years (13–15 yr) and the investment years (16–18 yr) as a function of sport [30]. Intervening categories are presented when data span across sampling and specializing years (e.g., 11–14 yr). As would be expected, practice hours increased across time. However, the so termed specializing and investment years showed little change across time in terms of average hours/week and hours/year of sport-specific practice. Soccer, volleyball and rhythmic gymnastics were the sports most represented in these figures. A few researchers also reported practice hours as a function of years into career and years of involvement [68, 74, 78], but due to the lack of studies, we have not included these data here.

**Figure 3:**
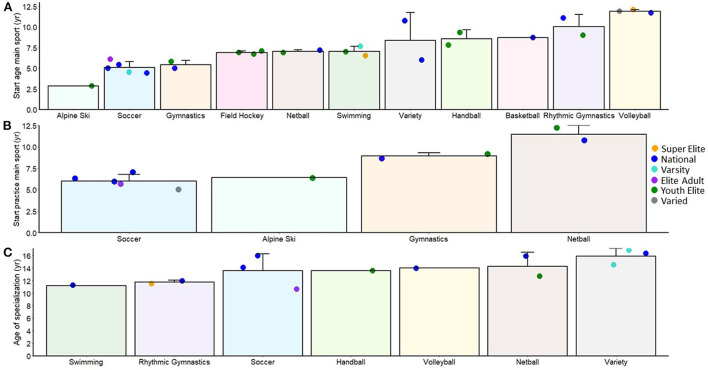
Sample (bar) and individual study means (colored circles) across different sports showing start age in the main sport **(A)**, start age in practice **(B)** and start age of specialization **(C)**. The sports are ordered in terms of start age and expertise category is shown in terms of a color coded grouping variable. Error bars show sample standard deviations.

In Figure 3, start age in the main sport (panel A), in main sport practice (panel B), and of specialization (panel C), as a function of sport and expertise, are shown. Data are shown as a function of sport and across the different skill groups given the range of start ages, which was sport and skill dependent. As can be seen in Figure 3A where the data are plotted in order of start age; alpine ski, soccer and gymnastics had early start ages before age 6 yr, whereas rhythmic gymnastics and volleyball had later start ages after age 10 yr. There were only a few sports that had multiple levels of expertise represented, but in general there were no skill-based trends across sports. In gymnastics, National athletes had slightly earlier start ages than Youth-Elite athletes, but this was reversed in rhythmic gymnastics. In soccer, youth athletes were not represented, but among adults, higher level athletes started soccer at an earlier age than the less elite groups. This earlier start age trend was also true for swimming, but here Youth-Elite athletes were represented and they had a slightly later start age than Adult Elite. Few studies reported start age in practice of the primary sport, but consistent with the overall start ages, alpine ski and soccer groups also began practice at a relatively young age (Figure 3B).

### Specialization and other sport participation

Eighteen studies included measures of practice and other sport participation and out of these, eleven studies also included reports regarding age of specialization. Specialization was defined as either exclusive engagement in one sport [33, 56, 66, 68, 69, 75, 76, 87] or age of investment without exclusive specialization [85]. As shown in Figure 3C, age of specialization was generally around 14 yr, with the exception of swimming and rhythmic gymnastics (~10–11 yr). There were a few sports that had data across multiple levels of expertise. In netball and soccer, both the Elite Adult (soccer) and Youth-Elite (netball) athletes specialized earlier than the National-level athletes, but there were no skill-group differences for rhythmic gymnastics.

**Figure 4:**
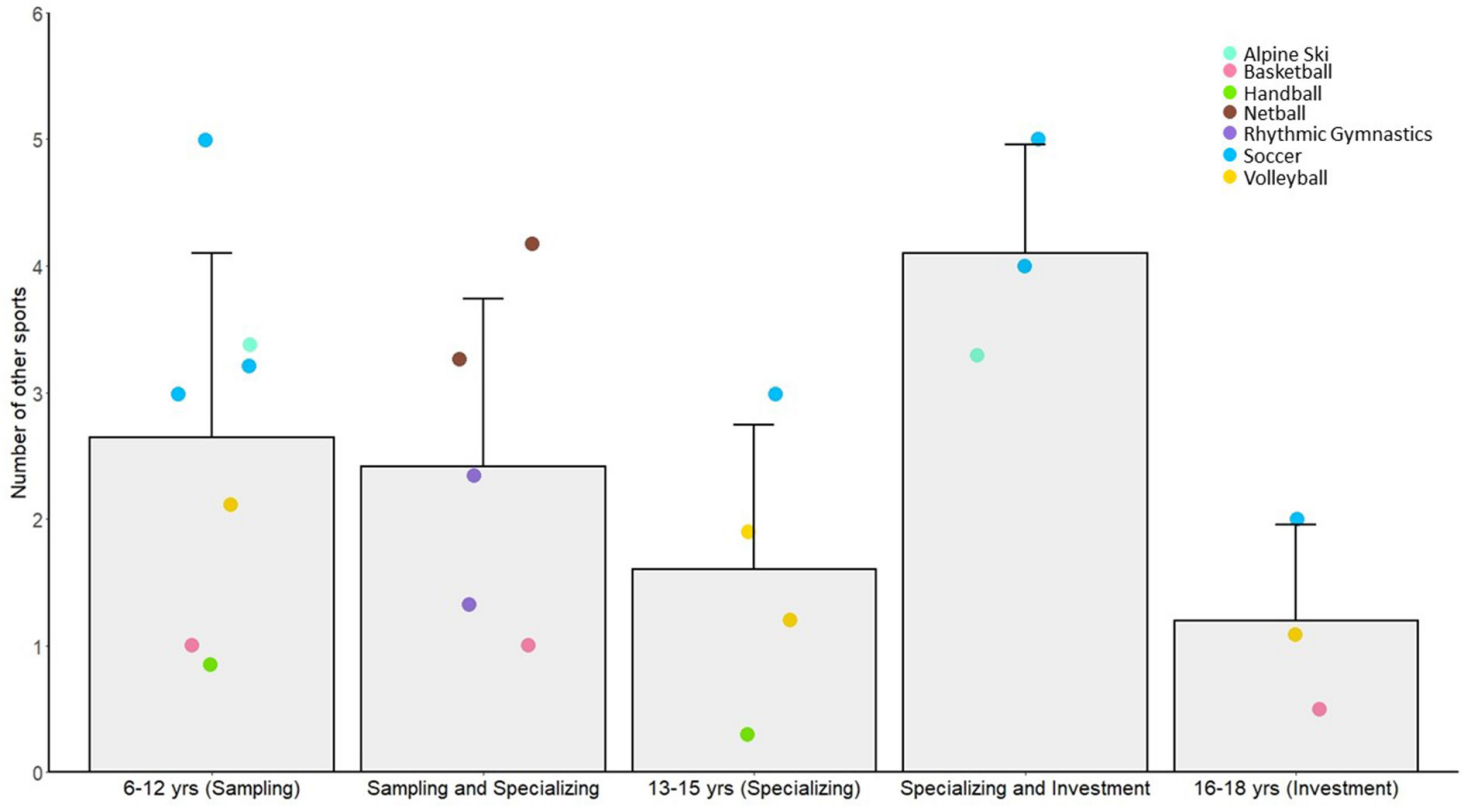
Sample (bar) and individual study means (colored circles) across different age categories defined in past literature as the sampling, specializing and investment years, for the average number of other sports participated by athletes. Error bars represent sample SDs and individual colored data points represent different sports and studies.

**Figure 5:**
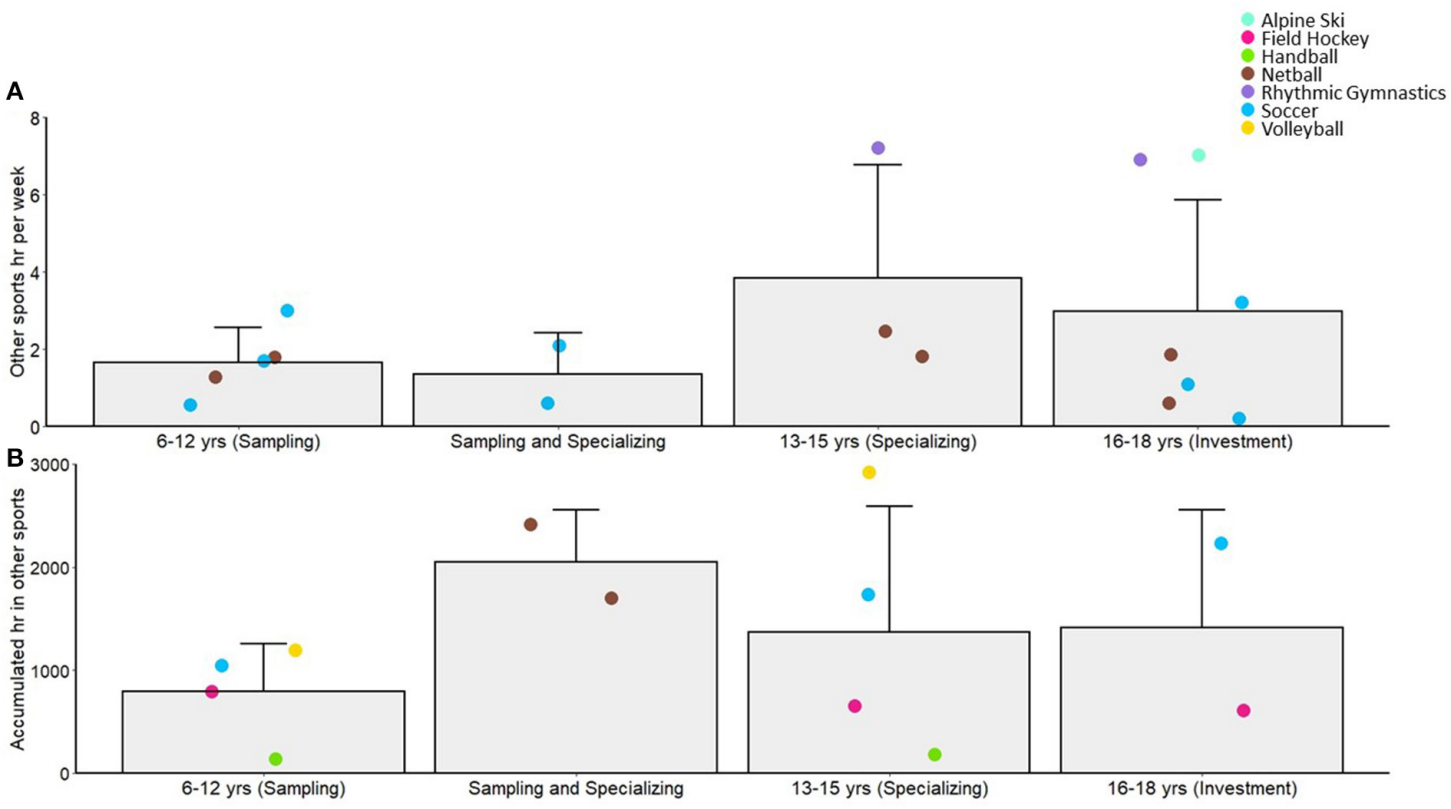
Sample (bar) and individual study means (colored circles) across different age categories defined in past literature as the sampling, specializing and investment years, for average hours per week in other sports **(A)** and average accumulated hours in other sports **(B)**. Error bars show standard deviation and colored circles represent individual groups for each sport/study. The “Sampling and Specializing” category represents data that span the ages in those categories.

The average number of other sports played within each age grouping is reported in Figure 4. Although there was a general drop off in sports from age 6–12 yr to 13–15 yr, this number increased for some sports (i.e., alpine ski and soccer) during the transition to the so termed “investment” years, what we have labeled specializing and investing (~15–16 yr). However, after the age of 16 yr the number of other sports was at its lowest. The number of hours per week and accumulated hours in other sports is presented in Figures 5A,B, respectively^2^. Again, there was a trend for increasing hours in other sports with age, rather than a decrease, especially in the so termed specializing/investment years (13–18 yr) as compared to “sampling” years (6–12 yr). Soccer and netball were the sports most represented showing these trends.

### Play amounts

**Figure 6:**
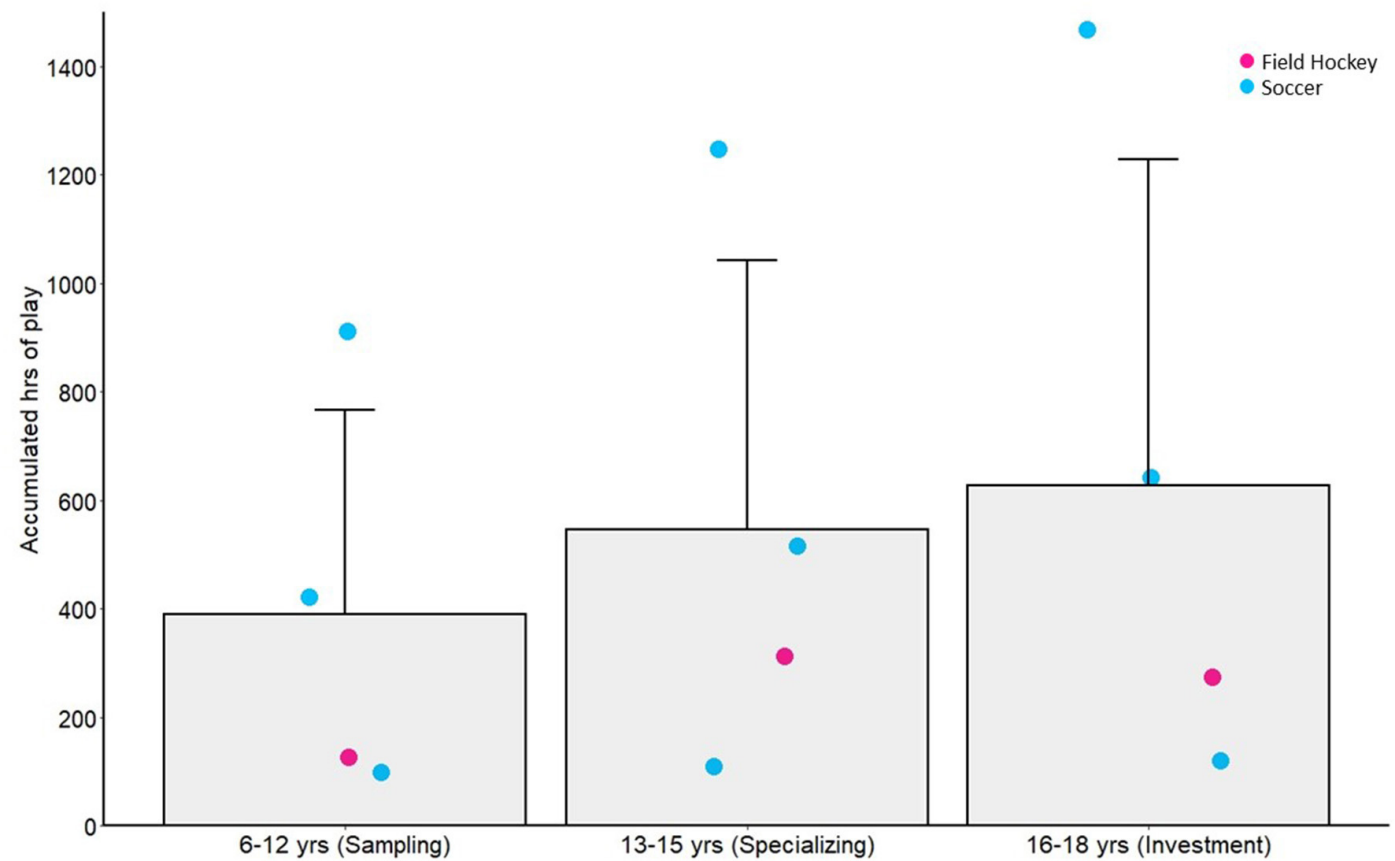
Sample (bar) and individual study means (colored circles) across different age categories defined in past literature as the sampling, specializing and investment years, for average accumulated hours of play. Error bars represent sample standard deviation and colored circles represent individual sport groups. The “Specializing and Investment” category represents data that span the ages in those categories.

Eight studies (representing two sports) included measures of play in the primary sport, or what were alternatively termed “unstructured activities” [59, 74, 75, 77, 86], as reported in Figure 6. Perhaps somewhat surprisingly, play activities continued to accumulate across development. Data from Youth Elite alpine skiers (age 15.7 yr) were not included [59], because hours/ year were only reported at one time point (~77 h).

## Discussion

### Included study characteristics

After an extensive literature search, we retrieved 32 studies representing 13 sports that quantified the practice, play, and specialization pathways of Adult Super Elite, National, Elite-Adult, Varsity, and Youth-Elite female athletes. Only swimming, soccer, volleyball, and rhythmic gymnastics had three or more levels of expertise represented, allowing some commentary on how these pathways differ across groups of elite athletes within a particular sport. In our sample, athletes competing at the adult National and Youth-Elite (national) levels were the most represented in terms of overall proportion of athletes and these athletes were distributed across a range of sports. Relatively few studies included Super Elite athletes (i.e., Olympic medalists), Professional (paid to play), Varsity, or Elite Club-level female athletes. This paucity in studies is somewhat expected for Super Elite athletes, as there are relatively few athletes competing at this high level, posing challenges for recruitment. In the case of female Professional athletes, fewer opportunities for paid play and the presence of a gender pay gap in sport [9] may contribute to the lack of research on these athletes. The lack of research on women University level athletes is more surprising. The sample heterogeneity present for sport and level of expertise makes it challenging to provide either general or comparative judgements about pathways to expertise for female athletes but we have nonetheless attempted to make some general conclusions. Identification of gaps in research with respect to sports, expertise, and outcome measures also allows some statement about directions for future research on women athletes.

Of the included studies, many (81%), reported measures of practice, although less than half (44%) reported measures of practice at multiple time points. In 59% of studies, multisport participation was quantified in some way, with sport specialization (31%) and play (28%) experiences detailed in about a third of the studies, potentially reflecting testable postulates of the DMSP [29]. All the included studies originated from Western countries, with large proportions originating from the USA, Portugal, Canada, and Australia. This geographical limitation is likely related to our search being restricted to those published with an English abstract. Environmental constraints, such as socio-cultural factors, have been proposed to interact with the development of expertise in sport [88, 89]. Because gender reflects how cultures and societies ascribe roles, characteristics and values to the sexes [18], gender constructs vary cross-culturally [90] and thus our conclusions about developmental pathways are unlikely to generalize outside of “westernized” contexts.

Measures of practice were often framed in the context of the DMSP or deliberate practice theory. There are numerous issues and debates surrounding the definition and subsequent ways of quantifying deliberate practice and potential (mis) interpretations of the definition of deliberate practice [19, 22–24]. Ericsson cautioned against conflating general measures of practice, play and competition with deliberate practice [19, 22]. However, this has been the norm in many sport-related studies, particularly in team sports, where the criteria for deliberate practice is unlikely to be met at the individual player level in group rather than individual practice settings. In our sample, researchers differed in how they qualified and quantified practice, with some distinguishing team and individual “deliberate practice” and others reporting total hours of general training. The majority of reports of practice were based on retrospective recall methods (78%; 30% retrospective interviews and 48% retrospective questionnaires), such that there may be tendencies for athletes to overestimate practice hours [91]. Over half of the studies included had measures of multisport participation. Some researchers defined other sport participation as regular participation for more than 1 month, while others did not define what constituted other sport participation. As also noted by Mosher et al. [36] there was considerable variability in how researchers defined and reported the number of other sport activities. In only a few studies were measures of play included and there was variability in how this was measured, with some including all sport-related play and others focusing specifically on play in the primary sport.

In the following paragraphs, we outline the general patterns of sport participation pertaining to the age categories detailed in the DMSP; that is ages 6–12 yr (so termed sampling), ages 13–15 yr (so termed specializing) and ages 16–18 yr (so termed investment years) [29, 30]. We consider the data with respect to evidence supporting a more diversified or specialized route for female athletes and relate these considerations to the early specialization pathway (which is more in alignment with deliberate practice theory), early majority engagement pathway [40], and the early diversification pathway of the DMSP. Where possible, we consider age and skill-based comparisons in view of a suggested dissociation in patterns of sport involvement that define junior and adult elite athletes [47].

### Early childhood (6–12 yr)

In general, the early childhood years were characterized by moderate engagement in practice activities with diversified sport participation. Athletes participated in an average of 2.7 other sports for 1.7 h/week, accumulating 788 h of practice in other sports during the sampling years. Although this is in line with the early diversification pathway of the DMSP [29, 30], there was also significant investment in sport-specific practice activities at a young age; with an average of 3.5 h of practice/week, 260 h of practice/year, and 927 accumulated hours of practice in the primary sport. Notably, the majority of time was spent practicing in the primary sport for these future elite athletes, even in these early childhood years, in line with the early majority engagement pathway [40, 41]. Although there were few studies where play amounts were reported, athletes reported relatively high amounts of play during early childhood (*M* = 390 h accumulated), which translated to ~33% of their sport time spent in play activities relative to practice.

The ages in which athletes began participating in their primary sport was highly varied in our sample, although this variability was mostly between sports rather than between categories of expertise. Within sports there were small differences in the start age in the primary sport across different categories of expertise. In gymnastics and swimming, the adult National and Super Elite athletes, respectively, had earlier start ages than the Youth-Elite groups. In soccer, although youth athletes were not represented, the higher-level adult athletes started earlier than the less elite. Although the data is lacking in women's and girls' sports, the current data does not show evidence supporting a delayed start age for adult elite athletes [47].

In sports such as gymnastics and figure skating, where peak success is often attained in late adolescence/early adulthood, our data were consistent with past research, where early specialized training is shown [10]. We also saw data consistent with ideas of late specialization sports, where coordination or physical requirements can hinder early engagement [10]. In our sample, several sports, including volleyball, field hockey, handball, netball, and rhythmic gymnastics, had quite late start ages compared to artistic gymnastics, soccer, swimming and alpine skiing (see Figure 3A). It is likely that these first mentioned ball control sports require developed motor skills and physical maturation for successful competition, leading to later sport starting ages. In the case of rhythmic gymnastics, there are coordination requirements that would need to be developed, in addition to fundamental gymnastic skills before athletes can start to use props and engage in this sport. Hence, our data on women and girls serve to further highlight the sport-dependent nature of pathways to expertise, necessitating sport specific recommendations in models of athlete development. These sport-specific data are consistent across male and female contexts because at young ages (before physical maturation), many sports offer mixed-sex/mixed-gender participation and sex differences in biological maturation do not emerge until adolescence.

In summary, elite female athletes engaged early in childhood in high amounts of sport-specific practice, whilst also engaging in approximately three other sports and high play amounts (where detailed). Their early childhood involvement would best be described as one of early majority engagement, rather than either early specialization or diversification.

### Early adolescence (13–15 yr)

In what has been termed the specializing years (i.e., 13–15 yr) [30], athletes devoted more time to their primary sport than in the sampling years, but without exclusive specialization. Practice hours were on average 14.8 h/week or 717 h/year. By the age of 15 yr, 2,023 h of practice had been accumulated on average. In these early adolescent years, there was increased variability in practice amounts both within and between sports, reflecting the unique training demands and constraints of the different sports. For the start age of specialization, there was again some consistency across sports and expertise categories, with the majority of sports showing specialization in this time period ~14 yr (with the exception of swimming and rhythmic gymnastics at ~11–12 yr). Soccer showed the largest range across skill groups (with specialization being reported as earlier for Elite Adult vs. Adult National athlete groups). These ages reported in this time period are mostly consistent with what would be expected based on the diversification pathway of the DMSP. Congruent with all developmental pathways, there was a decrease in the amount of time spent in play activities relative to practice during this period (21% of overall sport time).

Deviating from predictions of the early diversification pathway, the female athlete groups from our sample showed only a small reduction in the *number* of other sports in these so termed “specializing” years (*M* = 1.6), compared to the sampling years (*M* = 2.7) and actually increased the *hours* spent in these other sports by ~2 h from childhood (*M* = 3.8 h/week). Corroborating these data, 63% of a sample of National level soccer athletes participated in other sports in early adolescence [75]. However, not all researchers reported other sport participation in the early childhood years and as such the trends in the figures may be misleading [59, 80]. Youth-Elite alpine skiers only had data in late adolescence [59, 80] and National level rhythmic gymnasts did not have data represented during childhood [80]. For both these athlete groups, there was higher than average (>1 SD above the sample mean) other sport participation in adolescence [59, 80]. We do not know why other sport involvement would be so high for these groups, beyond the seasonal nature of skiing or potentially relatively lax definitions of diversified sport participation in these studies. Youth-Elite handball athletes participated in fewer (>1 SD below the mean) additional sports in the childhood and early adolescence years compared to the group mean [64]. Because these athletes specialized relatively late at a mean age of 12.7 yr (perhaps reflecting transitions to secondary school or high-school where sport-team practice is regulated by the schools on an almost daily basis), this may partially explain lack of involvement in other sports.

Supporting deliberate practice theory predictions, in both the childhood and early adolescent years, Super-Elite level (international medalist) rhythmic gymnasts accumulated more practice hours than both National-level rhythmic gymnasts and other sport groups (>1 SD above the sample mean) [33]. Similarly, groups of Youth-Elite and National-level gymnasts participated in more practice hours per week in gymnastics (>1 SD above the sample mean) than other sports in early and late adolescence, respectively [80]. Corroborating these data, a sample of figure skaters started practice at a younger age, but increased practice hours per week at a similar rate as team sport athletes and musicians, indicating that figure skaters were investing in their sport earlier than other groups [92] (note that these data were not included in our sample as the data were not disaggregated for gender).

### Late adolescence (15-18 yr)

Late adolescence, termed the “investment” years in the DMSP [30], was characterized by high amounts of practice (*M* = 13.9 h/week, 790 h/year, 4,508 h accumulated), but with continued participation in diversified sport activities (*M* = 1.2 other sports, 3.0 h/week). Following the predicted decline in play activities, these athletes spent 12% of sport time in play activities compared to practice in their primary sport.

National and Varsity adult soccer players and Youth-Elite alpine skiers participated in a relatively high number of other sports in the combined specializing and investment years (14–18 yr, *M* = 4.1 sports). In this category, groups maintained (Varsity soccer players and Youth-Elite alpine skiers) or slightly increased (National soccer players) the number of other sports in comparison to the sampling years [59, 77]. In male soccer athletes, diversified sport involvement has also been reported to be relatively high and maintained throughout development [42–45]. Moreover, the National soccer players represented in our sample had accumulated more hours of play in early and late adolescence compared to the group mean (>1 SD above the mean), although there were few sports represented with play amounts. National level volleyball players also continued to accumulate high amounts of play activities throughout the so termed specializing and investment years [57, 71]. This might reflect the cultural context for these athletes and the capacity for informal play activities in Portuguese volleyball.

### Limitations and recommendations

Although we were able to gather developmental data from over 40 groups of elite athletes, the interpretation and generalizability of our findings are limited in several ways. First, there was considerable heterogeneity across sports, across categories of expertise and concerning definitions and types of measures reported. As such, broad statements are difficult to make that represent pathways descriptive of girls and women athletes generally. On the positive, the diversity in sports and categories of expertise captured in this work does allow us to describe a broad range of athlete experiences, providing a strong base for future work. Although others have tried to aggregate across sports based on whether they are team or individual [e.g., [93]], or game sports vs. CGS (centremetre, grams or seconds) sports [e.g., [47]], this variation noted across sports in our review, even within those that might be considered to be of the same category (i.e., soccer and volleyball), illustrates what gets lost or misinterpreted through such aggregation. What we would like to see are more systematic investigations within specific sports amongst girls and women athletes, including longitudinal follow up studies, especially following those athletes who achieved success at the youth/junior levels of sport. This sport-focused investigation coupled with increased specificity in measurement and definitions, will allow for better recommendations about pathways which best engender success and allow for later aggregation of data once such sport-specific nuances are known [91].

In many of the studies in our sample, research questions were posed in the context of the DMSP to discern between groups following what is considered an early specialization or an early sampling pathway. As such, the way data are collected may be biased by the model (i.e., where dichotomous categories are searched for, such as “specializers” or “non-specializers,” or data are collected within specific age bands that correspond to an already assumed period of specialization). There have been suggestions to consider practice amount and issues concerning specialization in the context of biological maturation, such that key age ranges for determining practice hours within the primary and in other sports (or non-sport activities) would be different across the sexes [11]. Such considerations are consistent with the long-term athlete development model [10] where sex based physical maturation characteristics impact generally on “advice” to progress from a training for fun to training to win level of engagement within a sport. Ideally we would be collecting data from individual athletes concerning their age of pubertal onset, but such individual-based data does raise concern for data collection methods and perhaps underscores a need for physiologists to team up with skill acquisition specialists to best collect data on developmental progressions.

## Conclusions

In this scoping review of developmental pathways of elite female athletes, we show some differences in how expertise has been attained in comparison to general pathways proposed in the literature, based predominantly on male athletes. In general, women elite athletes reported increasing practice amounts as they continued in their sport throughout the childhood years but deviated from predicted pathways by continuing participation in other sports throughout adolescence, in what have been proposed to be the specializing and investment years [30, 32]. In addition to highlighting differences in pathways, we also highlight a gender gap in our knowledge of developmental pathways leading to expertise in girls' and women's sports. Although the current literature spans a range of sports, the relative paucity of research on female athletes means that there are still not enough data within specific sports and categories of expertise to draw conclusions regarding sport-specific pathways and differences between elite groups. In addition to further study of pathways toward expert performance of elite female athletes, we also recommend collection of data from non-western socio-cultural contexts, longitudinal data throughout the development years (particularly through the adolescence transition) and well-defined definitions of practice, play and specialization to allow better comparisons across studies. It may be that pathways and hence models of elite sport development need to be different for males and females, particularly when opportunities for professional careers are currently limited and where biological and psychosocial differences in maturation exist across sexes and genders. In future, researchers may wish to move beyond testing current dichotomous models of athlete development to explore the upper and lower limits of early engagement (i.e., a continuum of specialization), as well as discerning differential consequences for early patterns of engagement for long-term success and other measures of continued sport participation (such as injury, and psychological wellbeing). Exploring these patterns across defined male and female samples would give researchers and practitioners an evidence-base to create more nuanced athlete development models and programs that offer the next generation of female athletes the opportunity to safely grow, develop and flourish in their future sporting endeavors.

## Data availability statement

The original contributions presented in the study are included in the article/supplementary material, further inquiries can be directed to the corresponding author/s.

## Author contributions

CP performed the study search and data extraction which was audited by DH. All authors contributed to conception and design of the study. All authors contributed to writing and revisions of the submitted manuscript.

## Funding

This work was supported by a SSHRC (Social Sciences and Humanities Research Council of Canada) SPRI (Sport Participation Research Initiative) grant awarded to NH (grant # 003385-2017).

## Conflict of interest

The authors declare that the research was conducted in the absence of any commercial or financial relationships that could be construed as a potential conflict of interest.

## Publisher's note

All claims expressed in this article are solely those of the authors and do not necessarily represent those of their affiliated organizations, or those of the publisher, the editors and the reviewers. Any product that may be evaluated in this article, or claim that may be made by its manufacturer, is not guaranteed or endorsed by the publisher.

## Footnotes

^1^Coutinho et al. noted that they used the same participants for their 2016 and 2021 studies. In the 2016 study, age ranges of 8–12, 13–16, and 17–20 yr were reported whereas yearly measures were given from ages 6–12 yr in the 2021 study. Because of the duplicate reporting in the 8–12 yr age range, we only included measures from the 6–12 yr category from the 2021 study, representing a larger age range. We did not include data from Coutinho et al. [56] as the sample characteristics and our measures of interest were identical to those in Coutinho et al. [57]. Similarly, Fawver et al. [58], and Cowan et al. [59] used the same sample of participants as reported in De Couto et al. [[60]; confirmed via personal communication], such that any duplicate outcomes of interest reported in this work were not included in our final analyses. There was also replication of participants across studies by Johnson et al. [61] and Johnson et al. [62]. We have included both Johnson et al. [61] and Johnson et al. [62] in the characteristics of included studies but have omitted duplicated data.

^2^Bruce at al. [68] and Cowan et al. [59] reported hours of other sport participation per year. Values were divided by 52 to convert to a weekly value.
